# Magnitude of missed opportunities for prediabetes screening among non-diabetic adults attending the family practice clinic in Western Nigeria: Implication for diabetes prevention

**DOI:** 10.4102/safp.v62i1.5082

**Published:** 2020-08-20

**Authors:** Oluwaseun S. Ojo, Ademola O. Egunjobi, Adefemi J. Fatusin, Bolatito B. Fatusin, Odunola O. Ojo, Farouq A. Ololade, Patience A. Eruzegbua, Oluseyi A. Afolabi, Ayomiposi A. Adesokan

**Affiliations:** 1 Department of Family Medicine, Federal Medical Centre, Abeokuta, Nigeria; 2 Department of Family Medicine, Federal Medical Centre, Zamfara, Nigeria; 3 Department of Nursing, School of Nursing, Abeokuta, Nigeria

**Keywords:** prediabetes, diabetes, missed opportunity, missed diagnosis, primary care, primary care physicians, family practice clinic

## Abstract

**Background:**

For many decades, hypertension guidelines recommended dual-arm blood pressure measurement. However, this practice is poor in Nigeria and its significance is largely unidentified. Hence, this study was done to determine the point prevalence of inter-arm blood pressure difference and its relationship with hypertension and diabetes mellitus.

**Methods:**

A cross-sectional study was conducted among 214 respondents at the general outpatient clinic of a tertiary hospital in Nigeria. Demographic characteristics and anthropometric indices were obtained. Blood pressure readings were obtained through sequentially repeated measurements in respondents’ arms.

**Results:**

One-hundred and eighty-six respondents had complete data given a completion rate of 86.9%. Systolic blood pressure was higher on the right and left arm in 102 (54.8%) and 56 (30.1%) of the respondents, respectively. Diastolic blood pressure was higher on the right and left arm in 73 (39.2%) and 63 (33.9%) of the respondents, respectively. The overall prevalence of significant systolic inter-arm difference (≥ 10 mmHg) and diastolic inter-arm difference (≥ 10 mmHg) were 24.2% and 18.8%, respectively. Significant systolic inter-arm difference (*p* = 0.033) and diastolic inter-arm difference (*p* = 0.01) were significantly more among respondents with hypertension and/or diabetes mellitus.

**Conclusion:**

The blood pressure readings in both arms were different among the majority of the respondents, being higher on the right arm in many of them. The prevalence of significant inter-arm difference was high in the unselected primary care patients studied especially among patients with hypertension and/or diabetes mellitus. Blood pressure measurement in both arms should become a routine practice during initial patients’ visits in primary care.

## Introduction

Prediabetes is a high-risk state for diabetes, characterised by blood glucose levels that are higher than normal but not in the diabetic range. There is an increasing prevalence of prediabetes worldwide.^1,2,3,4,5,6,7,8,9^ Recent data have shown that in developed countries, more than one-third of adults have prediabetes.^1,2^ A high prevalence of prediabetes has already been established in the countries of the Middle East and Africa, where the prevalence has been described as ‘alarming’ or ‘dramatic’.^3^ In Nigeria, the prevalence of prediabetes that depends on the criteria and study population varies between and across studies.^4,5,6,7,8^ The prevalence of prediabetes in various Nigerian studies has been reported to be between 1.1% and 22.3%.^4,5,6,7,8^

The diagnostic criteria of prediabetes are fairly uniform across various international guidelines,^9,10,11,12,13,14^ except for the American Diabetes Association (ADA) lower cut-off of 5.6 mmol/L as opposed to 6 mmol/L for impaired fasting glucose (IFG).^10^ Prediabetes is a long prodromal stage that people with type-2 diabetes mellitus (DM) go through. It is often asymptomatic and, without treatment, one-third to half of people with prediabetes will progress to type-2 diabetes over 6 years.^15^ Prediabetes is also a predictor of cardiovascular disease (CVD).^9,10^ If the condition is left to progress to diabetes, the risk of CVD increases three- to fourfold.^10^

Scientific evidence suggests that people with prediabetes can delay or reverse the progression to type-2 DM.^15^ The Diabetes Prevention Program (DPP) demonstrated that in overweight adults with prediabetes, lifestyle modification including modest weight loss, dietary change and increased physical activity reduced the risk of developing type-2 DM by 58%, whilst drug intervention with metformin reduced the risk by 31%.^1^ Given the enormous health burden posed by prediabetes, especially in sub-Saharan Africa, and its favourable reversibility potential if detected, intervention in the ‘prediabetes’ stage of the disease to prevent progression to diabetes and its complications may be a sensible approach to reduce the already huge burden of DM.

The majority of people living with prediabetes and DM seek care in primary healthcare facilities^16,17,18^ and, by extension, the General Outpatient Department (GOPD) of teaching hospitals in Nigeria because of the weak primary healthcare system. Primary care offers the best opportunity to identify people at high risk of prediabetes.^18^ Screening for prediabetes meets most of the five criteria that define optimal conditions for screening for any disorder.^19^ However, there is strong evidence that screening for prediabetes is an underutilised resource in primary care. Many primary care physicians also lack the knowledge of the risk factors, diagnostic criteria, management and prevention of prediabetes.^20,21,22^ Based on the aforementioned factors, it is sensible to say that many patients with prediabetes may be missed in primary care. The situation will be even worse in a low resource setting like Nigeria, where there are limited consultation times because of the overwhelmingly heavy patient load and low doctor-to-patient ratios.^23^

The early identification of patients who may likely have prediabetes through risk factors for prediabetes can improve screening and reduce missed diagnoses of prediabetes amongst patients attending the primary care facilities. To date, there has been no study conducted in Nigeria and Africa on the magnitude of missed opportunities for prediabetes screening in routine primary care physicians’ visits. This study aimed to determine the incidence of missed prediabetes diagnoses and to describe the magnitude of missed opportunities for prediabetes screening amongst primary care patients attending the family practice clinic of a tertiary hospital in Western Nigeria using the guideline-recommended risk factors. This may be an important step in improving the rate of prediabetes detection and diabetes prevention interventions.

### Operational definitions

- *Prediabetes* was defined as not being diagnosed with prediabetes previously and not on treatment for diabetes but having IFG of 5.6 mmol/L – 6.9 mmol/L.^10^
- *Undiagnosed diabetes* was defined as diabetes not previously detected in patients having a fasting blood glucose (FBG) of ≥ 7.0 mmol/L.^10^
- *Risk factors of prediabetes* are factors or conditions the presence of which will positively increase the likelihood of an existing prediabetes. In this study, seven risk factors were identified from various guidelines on prediabetes^9,10,11,12,13,14^: age ≥ 45 years, body mass index (BMI) ≥ 25 kg/m^2^, waist circumference, hypertension, previous deliveries of big babies, gestational diabetes and family history of DM in first-degree relatives.
- *Missed diagnosis* was defined in this study as a non-diabetic patient who had been seen and discharged from the clinic by the doctor but was discovered to have prediabetes following an FBG test by the research team.
- *A missed opportunity* was defined in this study as the inability of the primary care physicians to screen a non-diabetic patient who was eligible for prediabetes screening based on any of the seven risk factors.

## Research methods

### Study design

This was a hospital-based cross-sectional descriptive study.

### Study setting

The study was carried out at the General Outpatient Clinic (GOPC) of a tertiary hospital in south-west Nigeria. Nigeria has a weak primary healthcare system. The frail state of Nigerian primary healthcare places a heavy burden on tertiary hospitals. This results in inversion of the pyramidal distribution of patients such that the majority of patients who should be cared for at primary care level are seen at the GOPC of tertiary hospitals. This makes the GOPC of a tertiary hospital in Nigeria a first contact facility for many patients.

### Study population

The study population consisted of adult male and female patients aged 18 years and over attending the GOPC of the hospital. A monthly average of 1168 patients was seen at this clinic.

### Exclusion criteria

The exclusion criteria for the study were the following:

- patients with previously diagnosed DM
- patient who were not previously diabetic but had been sent for a blood glucose test by the attending doctor
- patients who had had a blood glucose test done in the last year
- patients who used drugs that could affect glucose metabolism, for example, steroids, Vitamin C, B-blockers, thiazide diuretics
- pregnant women
- patients with a severe illness that would make it difficult to follow the study protocol.

**Sample size:** The sample size was calculated using the following formula^24^:

$$n=z^{2}pq/e^{2}$$ [Eqn 1]

The above formula was used in this study because the estimated population size (number of patients attending GOPD in a year) was more than 10 000.

*p* = 50% (because there was no previous study on the incidence of missed prediabetes diagnosis among primary care patients who had previously been seen by doctors)

*q* = 1–*p* = 50%. At 95% confidence level and precision level of 5%, *Z* = 1.96 and *e* = 0.05

*n* = 1.96^2^ × 0.5 × (1–0.5)/0.05^2^

*n* = 384.16.

However, to allow for missing data, the adjusted sample size (*n*1) using an attrition (*d*) of 10% was calculated using the formula:

$$\begin{array}{l} n1=n/(1-d)=384.16/(1-0.1)=384.16/0.9=426.8.\\ \text{This was approximated to }427 \end{array}$$ [Eqn 2]

### Sampling method

A systematic random sampling technique was used to select 427 subjects attending the GOPC over a period of one month. With a monthly average of 1168 patients, the sampling interval was (1168/427) = 2.74. Therefore, every third patient who had previously been seen by the clinic doctors at our GOPC and who met the selection criteria was enrolled into the study. Consent was obtained from patients in order of their arrival at the clinic. The first three patients were asked to select one from three folded ballot paper strips containing two ‘No’ and one ‘Yes’ option. Amongst the three participants, the one who selected the ballot paper containing ‘yes’ was selected as the first participant. After the selection of the first participant, every third consenting and eligible patient who came to the clinic was recruited. This process was repeated on subsequent days until the desired sample size was achieved.

### Data collection and procedure

The usual practice at our GOPC is for the clinic nurses to do the vital signs assessment at the nursing station before patients see the doctor in the consulting room. After the patients had been completely examined by the doctors working at the GOPC, they were called into another consulting room where the principal investigator and co-investigators of this study engaged them. The exclusion criteria were ruled out through taking a brief history, a physical examination and a review of patients’ case files. Consent was obtained from eligible patients.

Baseline demographic and clinical factors were obtained by the investigators through an interview using a pretested questionnaire (Appendix 1). The questionnaire consisted of four sections: socio-demographic variables, guideline-recommended risk factors, physical examination and investigation.

The socio-demographic information included age, gender, level of education, marital status, ethnicity and religion. The guideline-recommended risk factors comprised the seven risk factors that had been identified from various guidelines for prediabetes,^9,10,11,12,13,14^ which included age ≥ 45 years, BMI ≥ 25 kg/m^2^, waist circumference, hypertension, previous deliveries of big babies, gestational diabetes and family history of diabetes in first-degree relatives.

Missed opportunities for prediabetes screening in this study were defined based on seven risk factors that had been identified from various guidelines on prediabetes. A missed opportunity was defined in this study as the inability of the primary care physicians to screen a non-diabetic patient who was eligible for prediabetes screening based on any of the seven risk factors mentioned above.

Blood pressure (first and fifth Korotkoff sounds) was measured twice by using a standardised mercury sphygmomanometer, 3 minutes apart consecutively on seated participants after they had rested for 5 min. An appropriately sized cuff was placed on the right arm and pulse occlusion pressure was determined. The cuff was inflated to 20 mmHg above that pressure. Systolic blood pressure (SBP) and diastolic blood pressure (DBP) were measured at Korotkoff sounds I and V.^25^ The mean of each of these two measurements was used for the estimation of SBP and DBP. The standard definition of hypertension as SBP of ≥ 140 mmHg and/or DBP ≥ 90 mmHg or current use of antihypertensive medicines was used.^26^

Height (cm) and weight (kg) were measured to the nearest 0.1 cm and 0.1 kg, respectively, according to the standard guideline.^26^ Weight measurement was taken using a weighing scale, with the participant standing still in the middle of the weighing scale’s platform, without touching anything and with the body weight equally distributed on both feet. Height was measured using a stadiometer, with the participant standing straight, head in the horizontal plane (i.e. looking forward), heels together, arms to both sides, shoulders relaxed and wearing neither shoes nor head wear. Heels, buttocks, scapulae and occiput were against the vertical board of the stadiometer. The headboard of the stadiometer was then lowered onto the highest point of the head with enough pressure to compress the hair. The measurement was read visually at the same level as the headboard to avoid errors because of parallax.^26^ Body mass index was calculated and patients were stratified into underweight, normal, overweight and obese based on the BMI.^27^ Waist circumference was measured using a flexible measuring tape, with measurements taken halfway between the lower border of the ribs and iliac crest in a horizontal plane. A waist circumference greater than 88 cm for women and 102 cm for men was considered to be abnormal.^28^

After the blood pressure and anthropometric measurements were done, each respondent was counselled on the importance of undergoing a definitive test for DM. Each participant was given an investigation slip and informed to come to the GOPC in the morning of any clinic day according to their convenience within the study period for the definitive tests (FBG) using glucometer (Accu-check Advantage, Roche Diagnostics, Mannheim, Germany). To reduce loss to follow-up, the participants were reminded telephonically about their appointment for the FBG test a day prior to it.

**FIGURE 1:**
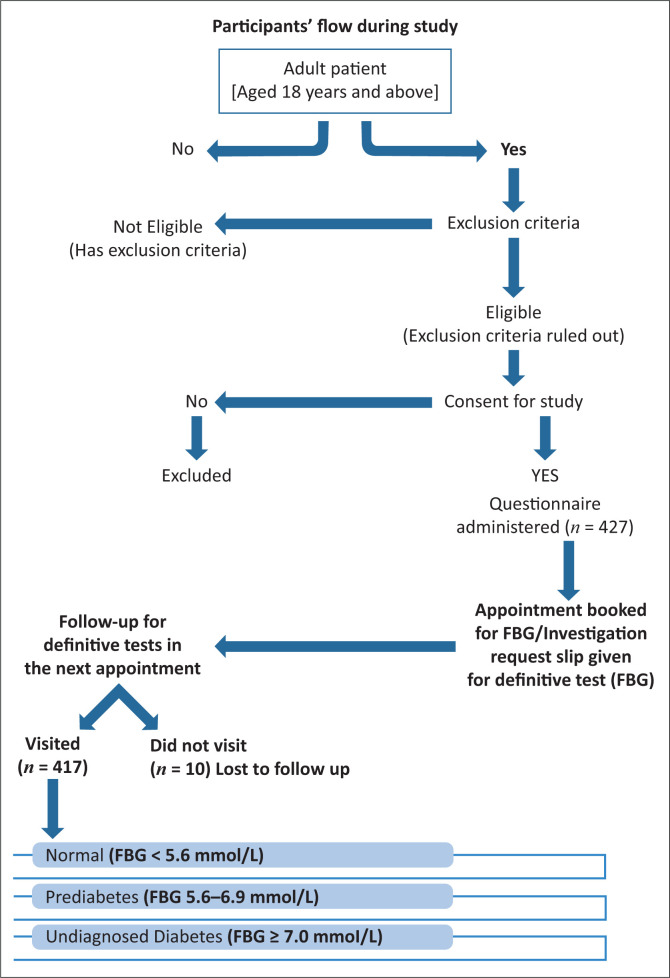
Participants’ flow during the study.

On the appointment day for the definitive test, FBG was measured by the investigators at the GOPC. The participant’s thumb fingertip was cleaned with an alcohol swab and the arm was allowed to hang down to let blood flow to the fingertip. The side of the thumb was pricked with a lancet and the drop of blood formed was sucked into the test strip. Blood glucose level was read from the glucometer screen. Respondents with an FBG of ≥ 7 mmol/L were diagnosed as having DM.^10^ Respondents with FBG between 5.6 mmol/L and 6.9 mmol/L were diagnosed as having prediabetes.^10^ Respondents with either prediabetes or DM were regarded as having dysglycaemia. Those who did not come for the FBG were considered as being a loss to follow-up. Those who had an FBG of ≥ 7 mmol/L (diabetes) were sent to the laboratory for a repeat test, which eventually confirmed DM. Newly diagnosed diabetic and prediabetic respondents were properly counselled regarding diets, exercise and adherence to medications, for those who needed antidiabetics. Based on the initial review, they were registered at the Family Physician-led GOPC or referred to the diabetologist (see Figure 1 for participants’ flow during the study).

### Duration of the study

The study was conducted over a period of 4 weeks.

### Data analysis

Data were analysed using the Statistical Package for Social Sciences (SPSS) version 22.0. The respondents’ characteristics (socio-demographic and clinical) were described using appropriate tables and charts. Continuous variables were summarised as mean and standard deviations, whilst categorical variables were summarised as percentages. The prevalence of missed prediabetes diagnosis was calculated. The proportion of respondents who missed the opportunity for prediabetes screening based on each risk factor was calculated. In addition, the proportion of respondents with multiple missed opportunities was also calculated. The proportions of missed prediabetes diagnoses based on the major criteria for screening and the number of risk factors were estimated.

### Ethical considerations

Ethical approval to conduct the study was obtained from the Research Ethics Committee of Federal Medical Centre Abeokuta, with protocol number FMCA/470/HREC/10/2016/30 and Federal Wide Assurance Number U.S./REG. NO.: FWA/00018660/02/28/2017. Informed consent was obtained from all the participants. A sample of the consent form is presented in Appendix 2.

## Results

Of the 427 respondents who were eligible and interviewed for the study, 10 were lost to follow-up and had missing data. Data analysis was performed for 417 respondents, which gave a completion rate of 97.7%.

The mean age of the respondents was 43.74 ± 16.32 years and 59.2% of them were women (Table 1 and 2).

**TABLE 1:** Socio-demographic characteristics of the respondents.

Variable	Categories	Frequency	
		*n*	%
Age	< 45 years	233	55.9
	45–54 years	68	16.3
	55–64 years	55	13.2
	≥ 65 years	61	14.6
Gender	Male	170	40.8
	Female	247	59.2
Marital status	Single	89	21.3
	Married (current or previous)	328	78.7
Religion	Christian	278	66.7
	Islam	136	32.6
	Traditional	3	0.7
Ethnicity	Yoruba	382	91.6
	Hausa	3	0.7
	Igbo	18	4.3
	Others	14	3.4
Level of education	No formal education	38	9.1
	Primary	76	18.2
	Secondary	115	27.6
	Tertiary	188	45.1

**TABLE 2:** Summary statistics of the continuous variables.

Variable	Minimum	Maximum	Mean ± standard deviation
Age (years)	18	90	43.74 ± 16.32
BMI (kg/m^2^)	14.47	42.51	24.00 ± 4.88
SBP (mmHg)	80	220	117.94 ± 19.02
Diastolic blood pressure (mmHg)	50	120	73.45 ± 12.68
Waist circumference (cm)			
All participants	57	136	89.17 ± 12.29
Male			87.85 ± 12.46
Female			90.07 ± 12.11
FBG (mmol/L)	4.00	11.61	4.94 ± 0.68

BMI, body mass index; SBP, systolic blood pressure; FBG, fasting blood glucose.

**FIGURE 2:**
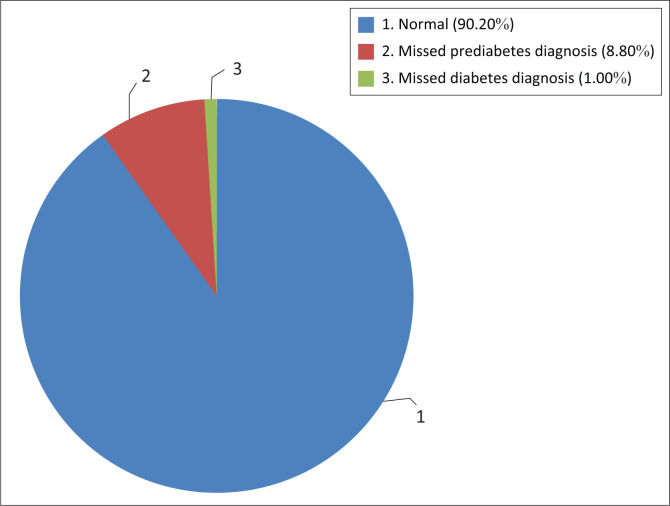
Incidences of missed prediabetes and diabetes diagnoses amongst respondents.

The incidences of missed prediabetes and diabetes diagnoses amongst the respondents were 8.8% and 1.0%, respectively (Figure 2).

The prevalence of missed opportunities for the prediabetes and diabetes risk factors ranged from 2.2% to 44.1% (Table 3).

**TABLE 3:** Magnitude of missed opportunity based on the guideline-recommended risk factors for prediabetes screening.

Variable	*n*	%
**Age**		
< 45 years	233	55.9
**≥ 45 years**	**184**	**44.1**
**Family history of diabetes**		
**Yes**	**174**	**41.7**
No	243	58.3
**Previous history of gestational diabetes**		
**Yes**	**9**	**2.2**
No	198	47.5
Not applicable	210	50.3
**Previous history of big baby**		
**Yes**	**41**	**9.9**
No	167	40.0
Not applicable	209	50.1
**BMI**		
< 25 kg/m^2^	269	64.5
**25–30 kg/m** ^2^	**98**	**23.5**
**≥ 30 kg/m** ^2^	**50**	**12.0**
**Waist circumference**		
Normal	277	66.4
**Abnormal**	**140**	**33.6**
**Hypertension**		
No	332	79.6
**Yes**	**85**	**20.4**

*Note:* The data set in bold indicates the respondents who had the various guideline-recommended risk factors for prediabetes

BMI, body mass index.

Amongst the respondents, 79.9% had at least one guideline-recommended risk factor for screening but were missed by the doctors (Table 4). The proportions of respondents who were missed based on the two major criteria recommended by ADA were 44.1% for age ≥ 45 years and 17% for age < 45 years with BMI ≥ 25 kg/m^2^ (Table 5).

**TABLE 4:** Proportion of missed opportunities based on number of guideline-recommended specific risk factors for screening.

Variable	Frequency	
	*n*	%
No risk factor	84	20.1
One risk factor	109	26.1
Two risk factors	83	19.9
Three risk factors	82	19.7
Four risk factors	38	9.1
Five risk factors	15	3.6
Six risk factors	6	1.5
Seven risk factors	0	0.0
At least one risk factor	333	79.9
No risk factor	84	20.1

**TABLE 5:** Magnitude of missed opportunity based on the guideline-recommended major criteria for prediabetes screening.

Variable	Category	Frequency	
		*n*	%
Age ≥ 45 years	Yes	184	44.1
	No	233	55.9
Age < 45 years with BMI ≥ 25 kg/m^2^	Yes	71	17.0
	No	346	83.0
Age > 45 years and/or BMI ≥ 25 kg/m^2^	Yes	254	60.9
	No	163	39.1

BMI, body mass index.

**FIGURE 3:**
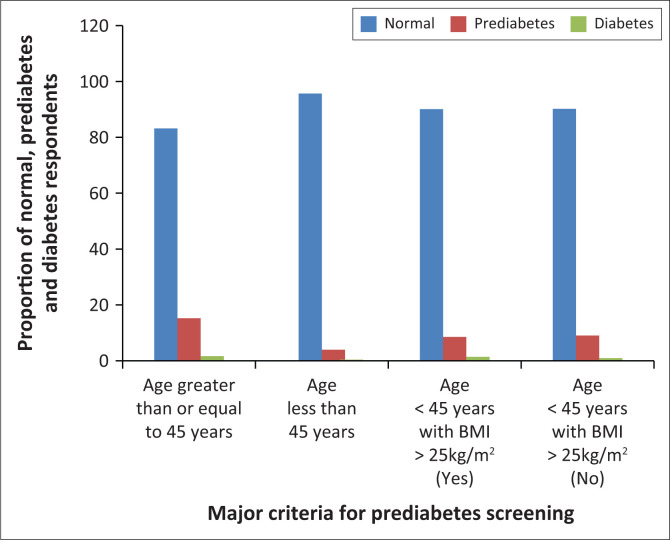
Proportion of missed prediabetes and diabetes diagnosis based on the major criteria.

**FIGURE 4:**
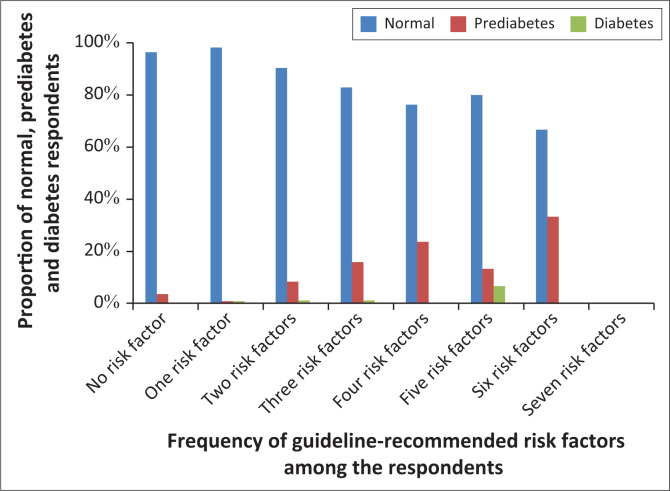
Proportion of prediabetes and diabetes based on the number of guideline-specific risk factors.

The proportion of missed prediabetes and diabetes diagnosis was higher in respondents with the major criteria (age ≥ 45 years and age < 45 years with BMI ≥ 25 kg/m^2^) when compared with their opposite (age < 45 years and respondents who do not belong to age < 45 years with BMI ≥ 25 kg/m^2^) (Figure 3). The higher the number of risk factors present in the respondents, the higher the proportion of respondents with prediabetes and diabetes (Figure 4).

## Discussion

To the authors’ knowledge, studies that have examined the magnitude of missed opportunities for blood glucose screening in routine primary care physicians’ visits are non-existent in Africa. The majority of the studies on prediabetes have centred on the burden of prediabetes in primary care and primary care physicians’ practice *vis-à-vis* diabetes evaluation and management.^1,2,3,4,5,6,7,8^ This study is, therefore, addressing an important gap in the literature.

The proportion of respondents with prediabetes who would have been missed by primary care physicians in this study was approximately 9%. Prediabetes in primary care is often unrecognised, with a consequent loss of opportunities for diabetes prevention. Whilst few studies have been conducted on this topic in developed countries,^19,22,29,30^ there is a paucity of studies on the magnitude of missed prediabetes diagnosis amongst primary care patients in Nigeria.

The general consensus of the few studies conducted on this topic is that primary care physicians still under-screen primary care patients for prediabetes and other chronic diseases.^19,22,29,30^ A study was conducted by Rieger et al.^19^ in the United States of America amongst 80 non-diabetic adult patients who were already flagged in the electronic medical record (EMR) for blood glucose screening because their BMI was greater than 25 kg/m^2^. This was done to improve screening for prediabetes by doctors. Despite EMR flagging, only 17 (21%) of the patients were screened by the doctors.^19^ Similarly, Bauer conducted a study on the predictors of missed opportunities for blood glucose screening amongst 274 church-affiliated African-Americans and found that, based on self-report, the proportion of those who were screened in the past one year was 54%.^29^

Based on the ADA protocol, which states that asymptomatic adults aged ≥ 45 years or < 45 years who have BMI ≥ 25 kg/m^2^ with additional risk factors should undergo prediabetes screening,^10^ this study showed that 44.1% and 17.0% of the respondents, respectively, met the criteria but missed the opportunities to be screened. Interestingly, amongst the respondents in this study who had prediabetes and/or diabetes, 75% of them were aged 45 years and over. It is also noteworthy that the primary care physicians in this study even failed to screen respondents who had four or more risk factors. Remarkably, the incidence of prediabetes in respondents with ≥ 4 risk factors was over 10%.

The lack of screening and detection exemplified in this study should be considered in light of the fact that most of these respondents have other cardiovascular risk factors, more importantly, hypertension (incidence of 20.4%), a common comorbid condition with diabetes. The poor cardiovascular outcome that is associated with co-existing dysglycaemia and hypertension underscores the importance of early detection at prediabetes stage to reverse the transition of prediabetes to diabetes.

Although our study did not investigate why primary care physicians do not often screen for prediabetes, potential explanations from the literature include poor knowledge about various published prediabetes screening guidelines^22,31^ and a lack of awareness of the potential effect of interventions in reducing diabetes.^29^ For some physicians, screening for prediabetes in patients with many comorbidities may not be regarded as a priority.^20,32^ Beyond the physicians’ inertia in screening patients, other barriers to screening include cost, transportation and mistrust of the healthcare system.^29^

The increased incidence of missed prediabetes diagnosis with age ≥ 45 years observed in this study is in agreement with previous studies on this topic.^6,7,8,33,34^ Age ≥ 45 years has been consistently shown to be associated with prediabetes and diabetes.^6,7,8,33,34^ Amongst the risk factors cited by various professional organisations for prediabetes and diabetes screening,^9,10,11,12,13,14^ the use of age as a screening tool will cost the physicians nothing in terms of time and energy. Despite its strong association with prediabetes and cost-effectiveness, it is still being used sparingly by primary care physicians.^29^ For example, 44.1% of respondents in this study met the criteria for screening (based on age), but these respondents were missed by the primary care physicians. An American study conducted by Bauer et al. also reported that 14% of respondents missed opportunities of being screened despite meeting the criteria for screening based on age.^29^

Over one-third (35.5%) of the respondents in this study missed opportunities for prediabetes screening despite having BMI ≥ 25 kg/m^2^. It must be pointed out that this study was conducted in a clinic where the clinic doctors had the opportunity to know the BMI because the weight and height of patients are routinely measured by the nurses as part of the vital signs before the patient sees a doctor. Based on the authors’ anecdotal experience, the situation may be worse in a typical primary care clinic seen in Nigeria and other developing countries, where basic instruments for vital signs assessment may not be available. This is similar to the finding in an American study where an EMR had flagged patients with BMI > 25 kg/m^2^ prior to seeing a doctor yet still reported only 17 (21%) of the 80 patients flagged as being screened for prediabetes.^19^ The higher proportion of missed prediabetes diagnoses in respondents with BMI > 25 kg/m^2^, when compared with respondents with BMI < 25 kg/m^2^ in this study, corroborated the findings of previous studies on this subject^7,35^ as well as various professional organisations’ screening protocols,^9,10,11,12,13,14^ which support the screening based on BMI.

Systematic reviews and meta-analyses have shown that BMI and waist circumference measurement were useful in predicting diabetes.^36,37^ However, whilst there is a high sensitivity for the waist circumference that may be attributed to increase awareness of the disease in the public, the specificity in diagnostic criteria necessary for clinical practice is low.^38^ Therefore, most national organisations base their screening protocols on BMI and have not included the waist circumference measurement in their protocols.^9,10,11,12,13,14^

Primary care physicians may find the risk factors, in particular, age ≥ 45 years and BMI > 25 kg/m^2^, to be a worthwhile starting point. The clinic nurses who measure vital signs can be helpful in this area by flagging any patient aged 45 years and above and/or BMI > 25 kg/m^2^ during vital signs assessment. The primary care physician clinic can also be equipped with point of care testing for blood glucose. These approaches may improve the rate of blood glucose screening amongst high-risk primary care patients. Interventions to overcome previously identified barriers to prediabetes screening guideline adherence should be implemented, whilst further research should be conducted to identify other barriers and interventions in order to overcome them.

This study has some limitations. The paucity of research on this topic made it difficult for the authors to calculate the appropriate sample size. Nonetheless, the sample size used has highlighted the magnitude of missed opportunities for prediabetes screening in resource-poor primary care settings. Whilst FBG is equally appropriate in screening for prediabetes,^10^ it would have been more appropriate if the oral glucose tolerance test, which is the gold standard, had been used. The use of capillary blood glucose may also be a limitation. However, it has been shown that photometric venous and glucometric capillary glucose estimations compare well with each other.^39^ The setting of the study at a GOPC at a tertiary hospital is not a typical primary healthcare setting. The practice of primary healthcare at this clinic cannot escape possible tertiary care influence. This could, therefore, have impacted the study findings. Other factors that could affect the blood pressure, for example, smoking, recent consumption of coffee and so on, may have had an impact on the blood pressure values obtained in this study.

## Conclusion

This study revealed that there are indeed missed opportunities for prediabetes screening in primary care. The finding that high-risk patients with prediabetes in our setting often missed opportunities to be identified through screening suggests that primary care physicians in our setting need to improve on the practice of prediabetes screening. There is a need to conduct similar studies in other primary healthcare settings in order to assess whether there is consistency in the findings.

## Appendix 1: Questionnaire

RESEARCH PROFORMA ON MAGNITUDE OF MISSED OPPORTUNITIES FOR PREDIABETES SCREENING AMONGST NON-DIABETIC ADULTS ATTENDING THE FAMILY PRACTICE CLINIC OF A TERTIARY HOSPITAL IN WESTERN NIGERIA – IMPLICATION FOR DIABETES PREVENTION

Hospital research ethics committee assigned number:

Protocol number:

Good day Sir/Madam,

Thank you for consenting to participate in this study. This research is about evaluation of the magnitude of missed opportunities for prediabetes screening amongst Nigerian primary care patients and its implications on prevention of diabetes. It will help us serve you better. Your cooperation is needed to truthfully answer the questions below. All information will be strictly confidential and it will take only a few minutes. Thank you.

Serial number.………………………………….

Hospital number.……………………………….

Date.……………………………………………….

### A SOCIO-DEMOGRAPHIC VARIABLES

1) Age .…………. years
2) Gender:   (i) Male   (ii) Female
3) Marital status:   (i) Single   (ii) Married   (iii) Divorced   (iv) Separated   (v) Widowed
4) Religion:   (i) Islam   (ii) Christianity   (iii) Traditional Belief   (iv) Others
5) Ethnic group:   (i) Yoruba   (ii) Hausa   (iii) Igbo   (iv) Others
6) Level of education completed by subject:   (i) No formal education   (ii) Primary   (iii) Secondary   (iv) Tertiary
7) What do you do for a living? .…………………………………………………………….
8) What is your average monthly earning ……………………………………………………

### B CLINICAL FACTORS

9) Do your family members (parent or sibling) have diabetes?   □ Yes   □ No
10) Have you ever been found to have high blood sugar during pregnancy?   □ Yes   □ No
11) Have you ever given birth to a large baby weighing 4.1 kg or more?   □ Yes   □ No

### C ANTHROPOMETRIC MEASUREMENT

12) Weight …………….
13) Height .…………….
14) BMI ………………
15) Waist circumference ……………….
16) Systolic blood pressure …………………
17) Diastolic blood pressure …………………

### D INVESTIGATION

18) Fasting blood glucose .……………….

## Appendix 2: Informed consent

Dear Sir/Madam,

I hereby seek your consent to participate in this research.

I am a doctor at the Department of Family Medicine, Federal Medical Centre, Abeokuta. I intend to evaluate the magnitude of missed opportunities for prediabetes screening amongst Nigerian primary care patients and its implications on prevention of diabetes. This may assist in improving the rate of prediabetes detection and diabetes prevention interventions.

If you consent, a questionnaire will be administered on you followed by a physical examination and blood tests. The procedure will last for about 20 min. The result of this project will be published in a journal.

Your participation is entirely of your own free will and you can withdraw from the study at any time you like without explanation. Refusal to participate in the study will not affect your treatment in any way. You have the right to refuse to answer any question you do not want to answer.

Please note that any information collected will remain confidential. Your name will not be attached to any published results. Kindly indicate your decision by signing in the space below.

Thank you.

Date and signature or thumbprint of participant

Date and signature or thumbprint of witness
